# Effectiveness of Short Implants Versus Long Implants With Sinus Floor Elevation in Patients With Atrophic Posterior Maxilla: A Systematic Review and Meta-Analysis

**DOI:** 10.7759/cureus.89103

**Published:** 2025-07-31

**Authors:** Abdulwahab T Alenezi, Meshari Alkandari, Mohammed Alkandari, Danah Alkhashan, Fahad Albakheet, Abdulaziz S Owayed, Abdulrahman H Jamaan, Ahmad Mathoud, Bader Alsulaili, Ahmad Alrashidi, Sayed A Alsaleh, Yousef Alajmi, Rashed Aldhafeeri, Abdullah Alsaffar, Turki Alharbi, Ahmed Abdelaziz

**Affiliations:** 1 Department of Dentistry, Saad Al-Abdullah Block 2 Polyclinic, Al-Jahra, KWT; 2 Department of Dentistry, Rumaithiya Polyclinic, Kuwait City, KWT; 3 Department of Dentistry, Rabia Polyclinic, Farwaniya, KWT; 4 Department of Dentistry, Jaber Al-Ali Block 2 Polyclinic, Al-Ahmadi, KWT; 5 Department of Dentistry, Almutlaa Polyclinic, Al-Jahra, KWT; 6 Department of Dentistry, Aldoha Polyclinic, Kuwait City, KWT; 7 Department of Dentistry, Sulaibiya Polyclinic, Al-Jahra, KWT; 8 Department of Dentistry, Saad Al-Abdullah Block 10 Polyclinic, Al-Jahra, KWT; 9 Department of Dentistry, West Hawally Polyclinic, Hawally, KWT; 10 Department of Dentistry, Oyoun Polyclinic, Al-Jahra, KWT; 11 Department of Dentistry, Siddeeq Polyclinic, Hawally, KWT; 12 Department of Dentistry, Farwaniya Specialty Dental Center, Farwaniya, KWT; 13 Department of Biostatistics, Faculty of Medicine Al-Azhar University, Cairo, EGY

**Keywords:** histomorphometry, long implants, short implants, sinus floor elevation, systematic review and meta-analysis

## Abstract

Maxillary sinus floor augmentation (MSFA) has become the standard technique, aiming to increase vertical bone volume to accommodate standard-length implants, typically 10 mm or longer, with a predictable treatment modality. Data have sparked interest in short implants for patients with atrophic jaws. This systematic review and meta-analysis aimed to evaluate the clinical results of short implants compared to long implants with sinus floor elevation. A systematic search of PubMed, Scopus, Web of Science, and the Cochrane Library was conducted from inception to June 2025 to identify randomized controlled trials (RCTs) comparing short implants with long implants and sinus floor elevation in patients with atrophic posterior maxillae. The primary outcome was the mean change in marginal bone loss. Secondary outcomes included rates of implant survival, biological complications, and prosthetic complications. A random-effects model was adopted to pool mean differences (MD) and odds ratios (OR), with 95% confidence intervals (CI). STATA MP version 18 was used for all statistical analyses. Seven RCTs comprising 393 patients and 474 implants were included. Short implants resulted in a significant reduction in marginal bone loss (MD = -0.26 mm, 95% CI: -0.43 to -0.09, *p* < 0.001; I² = 56.29, *p* = 0.04) and lower rates of biological complications (OR: 0.39, 95% CI: 0.18 to 0.85, *p* = 0.02; I² = 0.00, *p* = 0.92) compared to long implants with sinus floor elevation. Moreover, there was no significant difference between short implants or long implants with sinus floor elevation in terms of survival rates of implants used (OR: 0.96, 95% CI: 0.74 to 1.25, p = 0.76; I2= 0.00, p = 1) or prosthetic complications (OR: 2.18, 95% CI: 0.82 to 5.82, p = 0.12; I2= 25.62, p = 0.44). In conclusion, short implants (<8 mm) may offer an alternative to standard grafting implants. However, further long-term RCTs are needed to draw clear conclusions on survival rates.

## Introduction and background

Introduction

Reconstructing the posterior maxilla after long periods of tooth loss is often a challenge in clinical implant dentistry. Bone resorption and progressive maxillary pneumatization lead to insufficient bone height for rehabilitation [1]. Thus, atrophic jaw patients require further complementary surgical procedures before implant installation. Surgical options to overcome limited bone include using sinus grafts to add height and modified surgical methods for low-density bone [2].

Maxillary sinus floor augmentation (MSFA) became the standard technique that aims to increase the vertical bone volume to accommodate standard length implants typically ≥10 mm with a predictable treatment modality [1]. However, these augmentation procedures had significant drawbacks, such as increased patient morbidity, extended treatment durations, higher financial costs, and the risk of surgical complications, including sinus membrane perforation or graft failure [3].

The use of short implants is a less invasive alternative to bone augmentation, which is often ≤8 mm in length, avoiding the need for extensive grafting procedures and offering clinical advantages such as shorter treatment durations, reduced morbidity, and cost savings [2]. Shorter dental implants offer a promising treatment option, expanding access to implant therapy for more clinicians in challenging cases. Short- and mid-term data regarding short implants show high survival rates and few complications. However, concerns remain regarding increased crown-to-implant ratios and potentially higher biological and technical complication rates [4]. The advancement in the design of the implant and its surface roughness has improved recently, overcoming previous reservations.

A growing number of randomized controlled trials (RCTs) have caught interest in the short implants in atrophic jaw patients and whether this simplified procedure offers potential benefits in comparison to standard length implants supported by bone augmentation [2,3]. Therefore, this systematic review and meta-analysis was warranted to evaluate and compare the long-term clinical outcomes of short versus long dental implants with bone augmentation for posterior jaw rehabilitation.

## Review

Methods and materials

While conducting this systematic review and meta-analysis, we followed the guidelines of the Preferred Reporting Items for Systematic Reviews and Meta-Analyses (PRISMA) [5]. Additionally, we also followed the guidelines of the Cochrane Handbook for Systematic Reviews of Interventions [6].

Literature Search

We performed a comprehensive search in PubMed, Scopus, Web of Science (WOS), and the Cochrane Central from inception until June 2025. The search strategy included the following terms: (“Maxillary Sinus Floor Augmentation” OR “sinus lift” OR “sinus graft”) AND (“short implant” OR “ultrashort implant”) AND (“standard implant” OR “long implant”). We included only studies published in English. We also manually retrieved the references of the included studies for additional relevant publications. Table 1 depicts the detailed search strategy for each database.

**Table 1:** Search strategy for each database.

Database	Search Terms	Search Field	Search Results
PubMed	((Maxillary Sinus Floor Augmentation) OR (sinus lift) OR (sinus graft)) AND ((short implant) OR (ultrashort implant)) AND ((standard implant) OR (long implant))	Title and Abstract, English	130
Cochrane	((Maxillary Sinus Floor Augmentation) OR (sinus lift) OR (sinus graft)) AND ((short implant) OR (ultrashort implant)) AND ((standard implant) OR (long implant))	All Fields, English	55
WOS	((Maxillary Sinus Floor Augmentation) OR (sinus lift) OR (sinus graft)) AND ((short implant) OR (ultrashort implant)) AND ((standard implant) OR (long implant))	All Fields, English	194
SCOPUS	((Maxillary Sinus Floor Augmentation) OR (sinus lift) OR (sinus graft)) AND ((short implant) OR (ultrashort implant)) AND ((standard implant) OR (long implant))	Title, Abstract, Keywords, English	152

Eligibility Criteria

We considered all records on their titles and abstracts initially, and those relevant to our eligibility criteria were then subjected to full-text screening to assess the pre-defined criteria. We included relevant studies if they met the following inclusion criteria: P): patients undergoing dentistry implantation; I) short implants defined as less than 8mm; C) standard length implants defined as 8-15mm in addition to sinus floor elevation; O) reported relevant outcomes using an intention-to-treat analysis; and S) randomized controlled trials (RCTs) only. On the other hand, we excluded studies that did not examine the short implants or the addition of sinus floor elevation during short implants as their rehabilitation procedures, as well as those with unpublished data, conference abstracts, or observational designs.

Outcomes

The studied primary outcome was the mean change of the marginal bone loss, with lower bone loss indicating a favorable outcome [7]. Additionally, other secondary outcomes were survival rates of the implants used and biological and prosthetic complications.

Quality Assessment

Two of the assigned authors independently adopted the Cochrane Risk of Bias 2 tool (ROB-2) to assess the risk of bias of all included studies [8]. The tool assesses five different domains of bias, including the following: selection bias via the randomization process, detection bias as outcome measurements, performance bias as deviation from intended interventions, attrition bias as missing outcome data, and reporting bias as selection of the reported results. The decisions were labelled as “high risk of bias”, “some concerns”, and “low risk of bias”. If there were any disagreements between the two assigned authors, a third author was needed to resolve the conflict.

Data Extraction and Meta-analysis

We used a manual Excel sheet to extract the needed data from the included studies. The data extracted were four domains: (1) summary of the included studies, including study design, country, follow-up duration, patient and implant numbers, implant length of intervention and control, prosthetic type, and findings of each study; (2) characteristics of the included patients, including sample size of each arm, the mean age, percentage of males, residual bone height, and the sinus lift approach; (3) risk of bias domains; and (4) measurements of the studied outcomes. Continuous data were extracted as mean and standard deviation (SD) reported from each study at the latest follow-up duration. Additionally, continuous outcomes were pooled as mean difference (MD) with its 95% CI using the Der-Simonian-Laird random-effect model. The heterogeneity was assessed using the Cochrane Q test, and the I2 measure was determined across all studies. A p-value less than 0.05 and an I2 value ≥50% were deemed as significant diversity among the included studies. The package “meta esize” and “meta forest plot” were used on STATA 18MP software to pool the effect estimate and the corresponding 95% CI. According to Egger’s test, publication bias is not reliable for less than 10 pooled studies [6]. Accordingly, in our study, we could not assess the existence of publication bias by Egger’s test for funnel plot asymmetry. Nevertheless, forest plots were reported.

Results

Search Results

**Figure 1:**
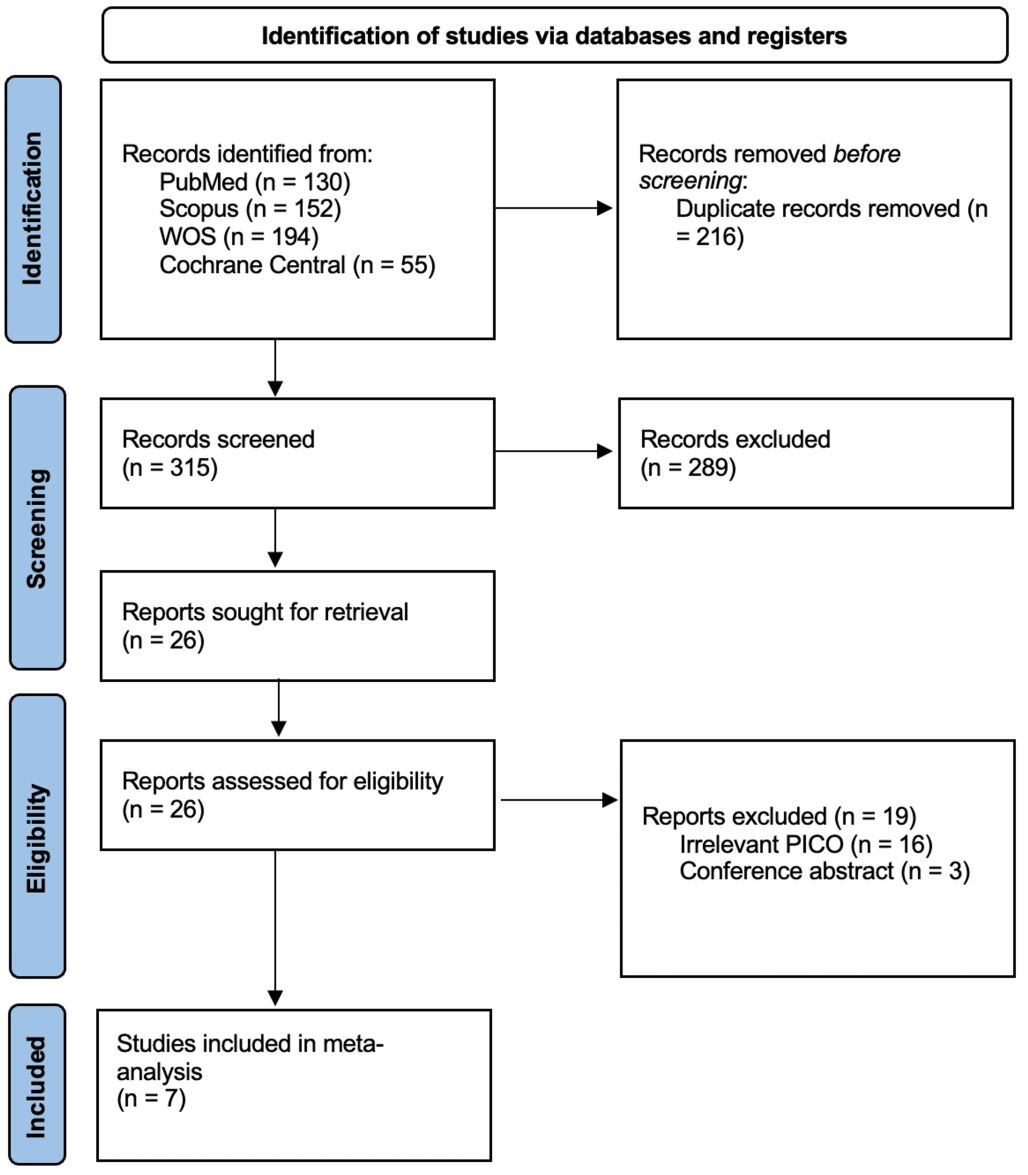
PRISMA flowchart of study selection process

We had a total of 531 citations from electronic databases, of which a total of 216 articles were excluded after the removal of duplicates, and a total of 7 RCTs were included in the final analysis after title, abstract, and full-text screening [1-4, 9-11]. A detailed selection process summary is found in the PRISMA chart (Figure 1).

Characteristics and Quality Assessment of the Included Studies

A total of seven RCTs comprising 393 patients and 474 implants were included in the final analysis, of which 235 implants (49.57%) were allocated to short implants, while 239 implants (50.43%) were allocated to long implants with sinus lift elevation. The mean age of the included patients was 54.3 ± 11.8 years, with 142 (36.13%) of the patients being females. Other detailed information on summary and baseline data of the included studies and patients is found in Tables 2-3. 

**Table 2:** The summary characteristics of the included studies.

Study ID	Country	Study Design	Patients, n, implants, n	Intervention	Control	Prosthetic Type	Conclusion	Follow-Up Period
Thoma et al., 2024 [4]	Switzerland, Austria, Poland, Spain, USA	RCT multicenter	61 patients, 86 implants	6 mm-long implants	Standard-length (11-15 mm) implants with sinus grafting	Single, non-splinted crowns	Good 10-year survival rates, reduced morbidity, and lower costs in the short implants group compared to longer implants with sinus grafting.	10 years
Felice et al., 2019A [9]	Italy	Split mouth RCT	40 patients, 72 implants	Length 6 mm, diameter 4 mm	Implants ≤10 mm, diameter 4 mm + sinus lift	Cemented	Short implants achieved similar results compared to long implants with sinus lift	5 years
Esposito et al., 2019 [10]	South Korea, Italy	RCT multicenter	72 patients, 73 implants	Length 5 mm, diameter 6 mm	Implant 10–15 mm plus sinus lift	Screw retained/cemented	Short implants achieved similar results compared to long implants with sinus lift	5 years
Felice et al., 2019B [11]	Italy	Split mouth RCT	81 patients, 83 implants	One to three 6-mm-long, 4-mm-wide implants	At least 10 mm long in augmented bone	Splinted (rigidly joined), cemented metal-ceramic or metal-resin fixed partial dentures	Short implants achieved similar results compared to long implants with sinus lift	5 years
Guljé et al., 2023 [1]	The Netherlands	RCT	41 patients, 41 implants	6 mm-long implants	One 11 mm-long implant placed with maxillary sinus floor	Single cemented zirconia-based porcelain crowns on titanium abutments	Both are equally successful during a 10-year follow-up period	10 years
Pohl et al., 2017 [3]	Austria	RCT	76 patients, 97 implants	6 mm-long implants	11-15 mm long implants with simultaneous sinus grafting	Single, non-splinted crowns (cemented or screw-retained)	Short implants achieved similar results compared to long implants with sinus lift	3 years
Durrani et al., 2024 [2]	India	RCT	22 patients, 22 implants	Implants ≤8 mm	Implants (>8 mm) with sinus augmentation	Single screw-retained porcelain-fused-to-metal (PFM) crowns	Short implants provided similar clinical and radiographic performance compared to long implants with sinus augmentation up to 12 months after prosthetic loading.	1 year

**Table 3:** The characteristics of patients included in the studies.

Study ID	Arms	Sample, n	Age		Gender (n) Male/Female	Residual Bone Height	Sinus Lift Approach
			Mean	SD			
Thoma et al., 2024 [4]	Short implants	39	50	14.05	21/29	5-7 mm	Lateral window
	Long implants	47	51	12.8	28/23		
Felice et al., 2019A [9]	Short implants	34	NA	NA	NA	4 to 6 mm	Lateral window
	Long implants	38	NA	NA	NA		
Esposito et al., 2019 [10]	Short implants	36	NA	NA	NA	5-7 mm	Lateral window
	Long implants	37	NA	NA	NA		
Felice et al., 2019B [11]	Short implants	39	57.6	NA	11/9	5-7 mm	Lateral window
	Long implants	44	57.6	NA			
Guljé et al., 2023 [1]	Short implants	21	50	10	7/13	6 mm	Lateral window
	Long implants	20	48	12	11/7		
Pohl et al., 2017 [3]	Short implants	47	50	14.05	21 / 29	5-7 mm	Lateral window
	Long implants	50	51	12.8	28 / 23		
Durrani et al., 2024 [2]	Short implants	11	NA	NA	NA	5-7 mm	Osteotome sinus floor, lateral window approach
	Long implants	11	NA	NA	NA		

**Figure 2:**
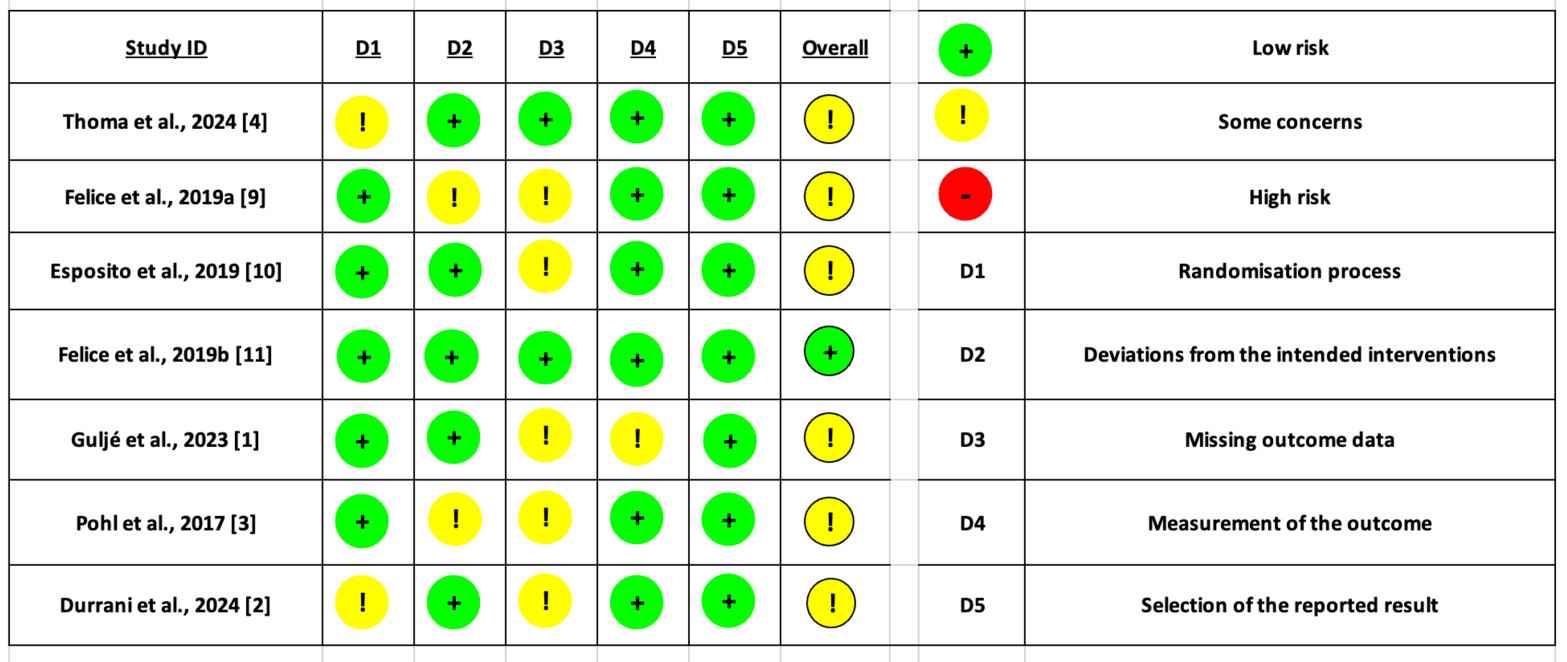
Risk of bias assessment using ROB-2 tool for RCTs. The majority of the included studies showed some overall concerns, mainly due to bias in the randomization process or bias due to missing data. While only one study showed an overall low risk of bias.

Regarding the risk of bias assessment using the ROB-2 tool, A total of six studies showed some overall concerns, mainly due to the randomization process and missing data. Moreover, only one study had an overall low risk of bias (Figure 2).

Outcomes

**Figure 3:**
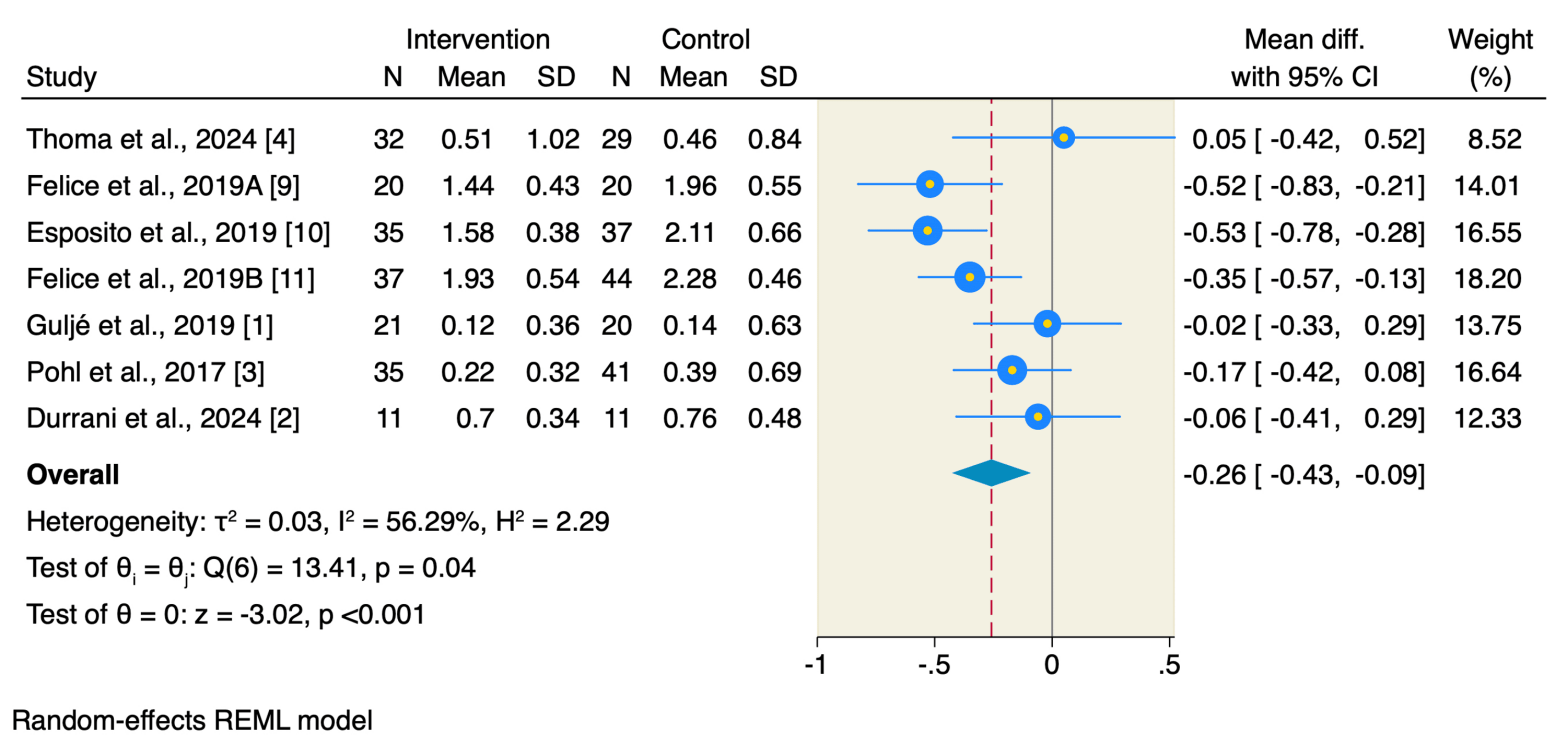
Forest plot of marginal bone loss. The marginal bone loss was reported in all included studies, and the pooled analysis showed that the short implants were associated with significantly lower marginal bone loss compared to longer implants with sinus floor elevation [1-4, 9-11].

All included studies assessed the marginal bone loss, of which short implants resulted in a significant reduction of marginal bone loss compared to long implants with sinus floor elevation (MD= -0.26 mm, 95% CI: -0.43 to -0.09, p < 0.001; I2= 56.29, p = 0.04) (Figure 3).

**Figure 4:**
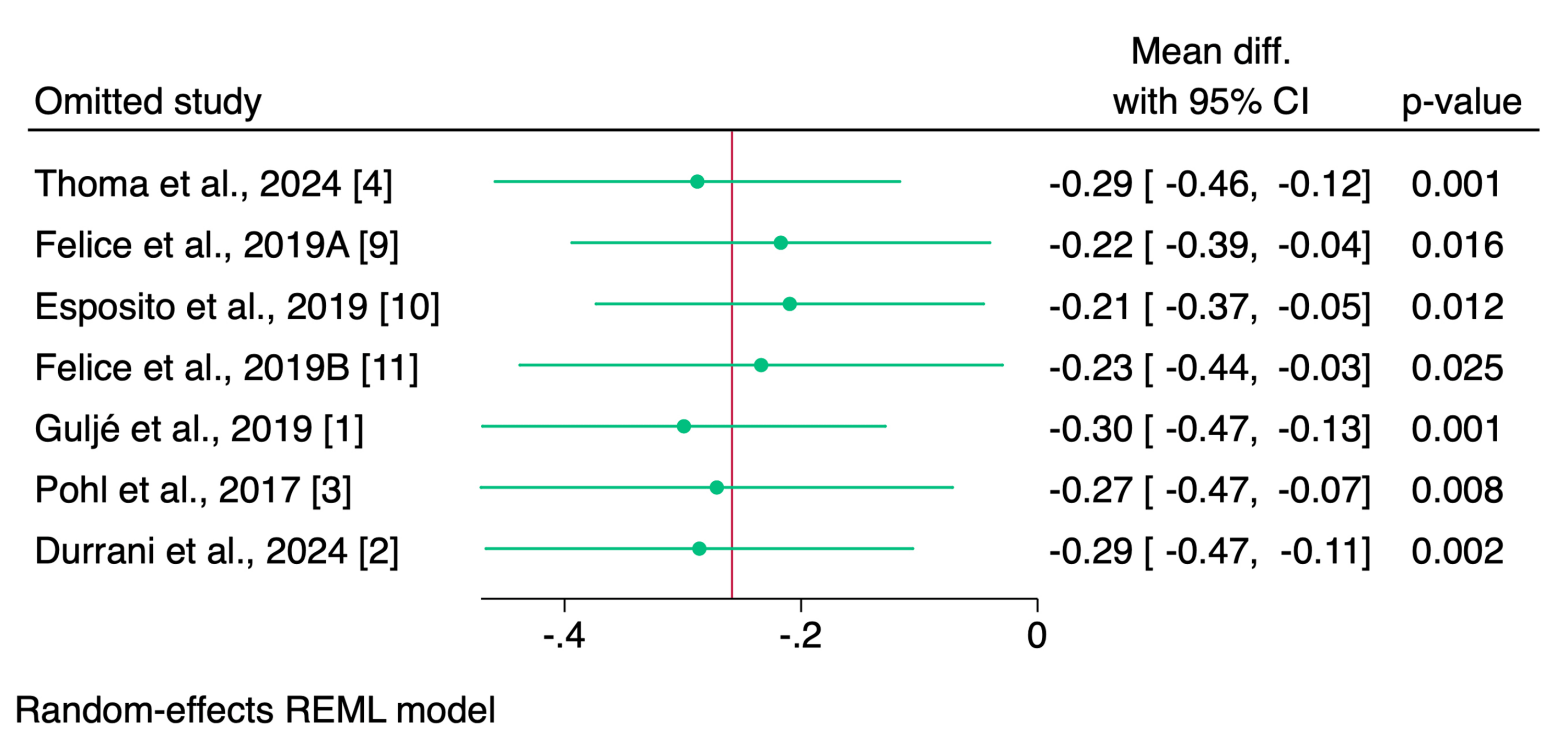
Leave-one-out sensitivity analysis plot of marginal bone loss. The leave-one-out sensitivity analysis showed that all omitted studies resulted in significant results, of which the results of the short implants were consistent across all included studies [1-4, 9-11].

Leave-one-out sensitivity analysis showed no single study had a disproportional effect on the overall effect estimate, confirming the superiority of short implants over long implants with sinus floor elevation (Figure 4).

**Figure 5:**
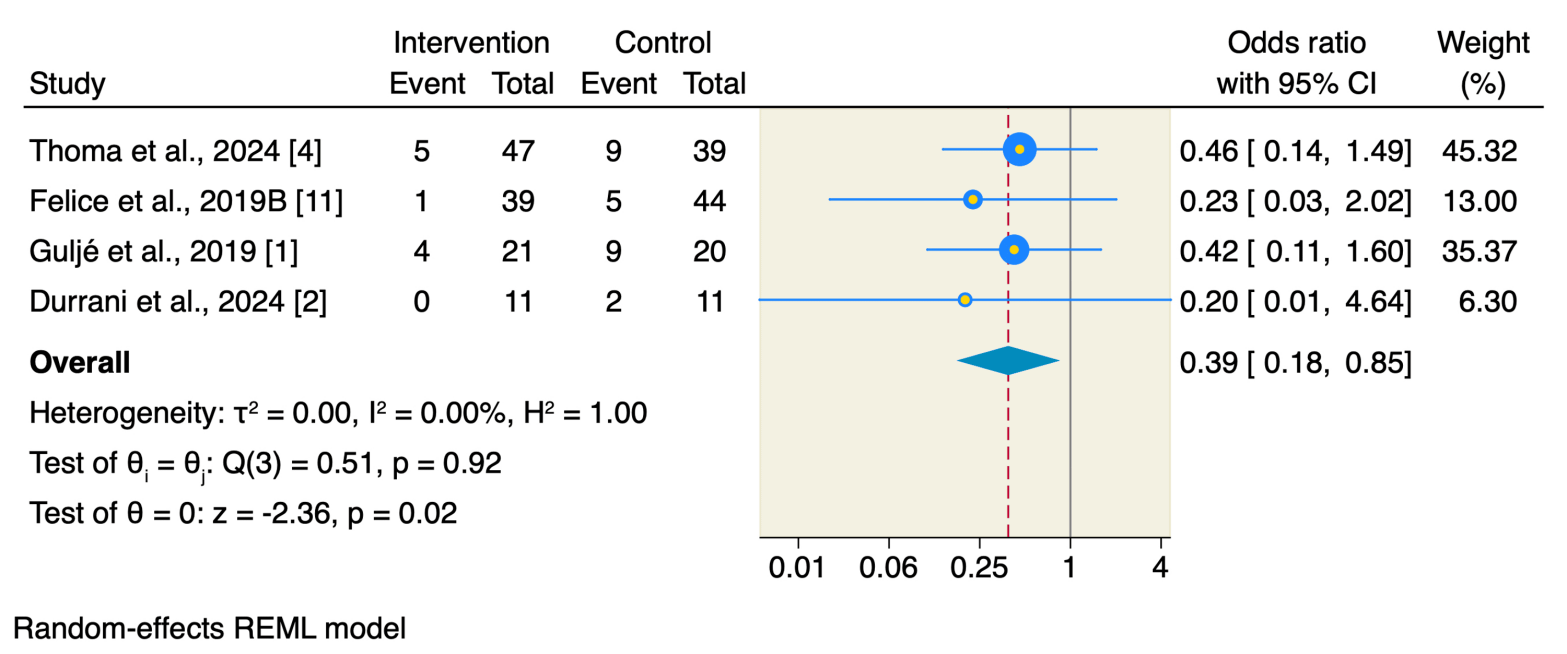
Forest plot of biological complications. The rates of the biological complications were reported in four studies, of which the pooled analysis showed that the short implants were associated with significantly lower rates of complications compared to longer implants with sinus floor elevation [1,2,4,9].

Additionally, short implants were associated with significantly lower rates of biological complications compared to long implants with sinus floor elevation (OR: 0.39, 95% CI: 0.18 to 0.85, p = 0.02; I2 0.00, p = 0.92) (Figure 5).

**Figure 6:**
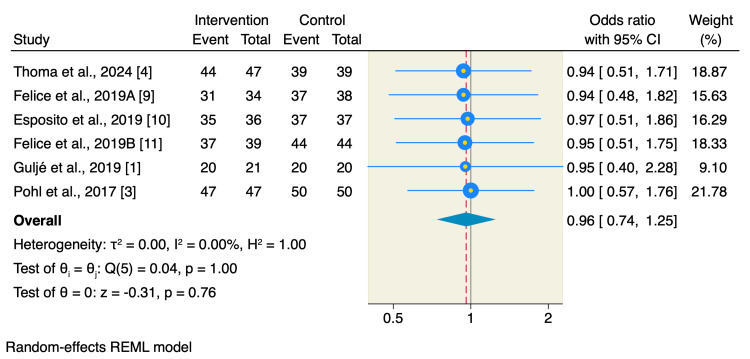
Forest plot of survival rates of implants used. The survival rates of the implants used were not significant between the two studied interventions, highlighting that both interventions resulted in similar survival rates [1, 3, 4, 9-11].

**Figure 7:**
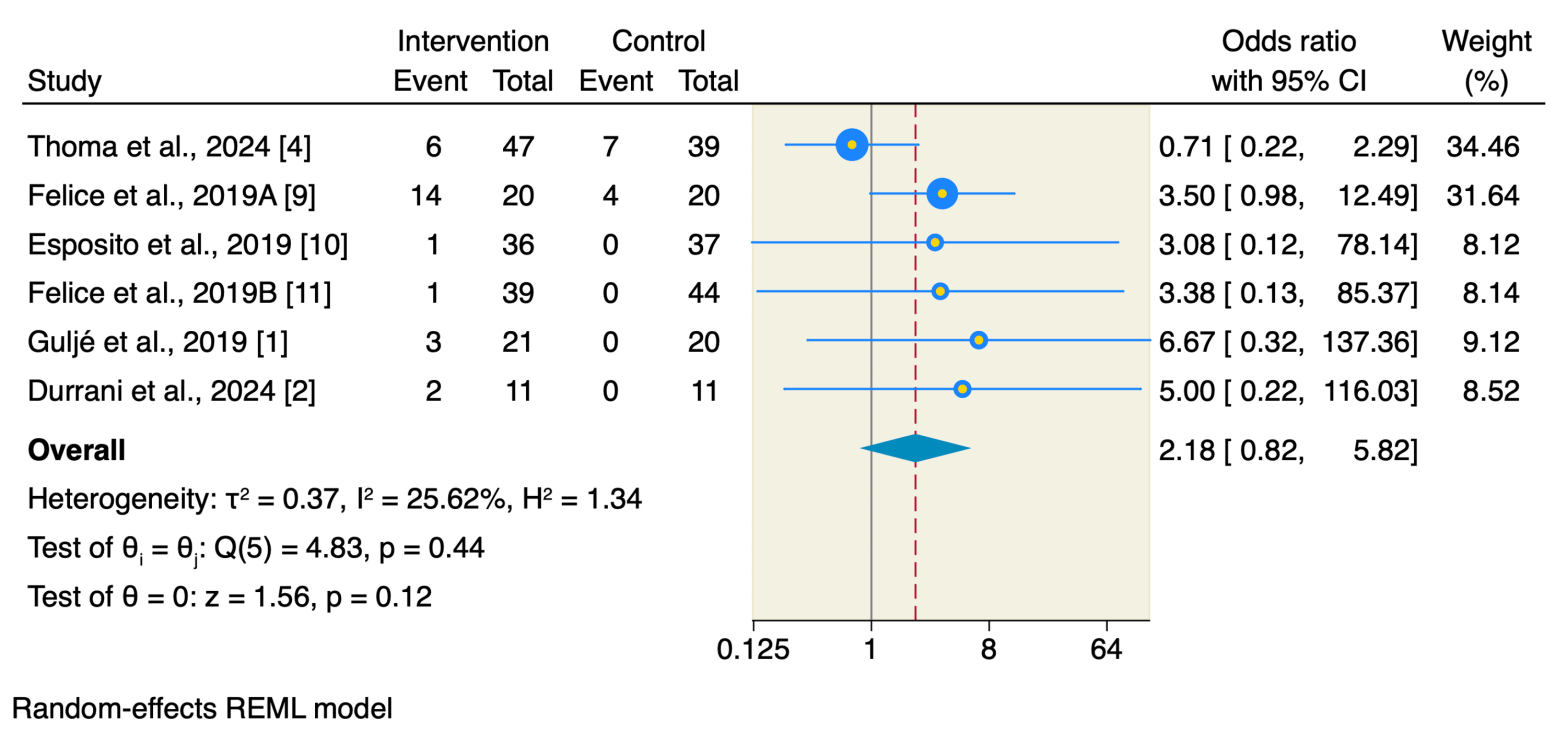
Forest plot of prosthetic complications. The rates of the prosthetic complications showed no significant difference between the two studied interventions, despite the higher trend in the short implants. [1,2,4,9-11]

Moreover, there was no significant difference between short implants or long implants with sinus floor elevation in terms of survival rates of implants used (OR: 0.96, 95% CI: 0.74 to 1.25, p = 0.76; I2= 0.00, p = 1) or prosthetic complications (OR: 2.18, 95% CI: 0.82 to 5.82, p = 0.12; I2= 25.62, p = 0.44) as shown in Figures 6-7.

Discussion

This systematic review and meta-analysis of seven RCTs and 393 patients and 474 implants is the most comprehensive meta-analysis to date, to assess the clinical parameters related to the effectiveness of using short implants in comparison with standard grafting with long implants, in addition to sinus floor elevation. Our study reported that short implants resulted in a significant reduction in marginal bone loss and biological complications compared to standard grafting without notable differences in other studied outcomes.

The use of long implants with sinus floor elevation has been considered the gold standard in patients with reduced bone height due to previous reporting of high implant survival rates [12]. However, in the past years, short implants were used for various clinical indications, which opened the door to assessing their efficacy and clinical implications compared to long standard grafting [13].

We reported significant reductions in the marginal bone loss in the short implants group compared to the longer implants group. The changes in marginal bone loss are viable in assessing the success of implant therapy. In addition, maintaining a stable marginal bone is a critical aspect of clinical success [14]. Thoma and his colleagues reported a 10-year follow-up study comparing short and long grafts and concluded that the mean marginal bone loss changes over the 10-year follow-up duration were minimal, indicating that the levels of bone loss were closely related to the implant shoulder, and this aligns with previous clinical trials that used the same implant system [4,15].

However, other RCTs concluded a significant reduction of marginal bone loss following grafting with short implants [9,10]. The major differences between the included studies are in the implant system, the available bone height, the location of the implant, either mandible or maxilla, and the material of the restorations. Additionally, the implants used across most of the included studies had a platform-switching connection that has shown a significant marginal bone loss with a butt joint connection [16]. Of note, the addition of the microthreaded design of the implants at the uppermost section may have contributed to the improved marginal bone loss [17].

Moreover, the mandible is shown to have higher implant loss compared to the maxilla, and the implant system and type, whether it is a one-piece implant or a two-piece implant, were the most relevant factors across studies [17].

Additionally, the rates of biological complications, such as peri-implant mucositis, varied across studies, with a significant overall reduction in the short implants group. The rates of the biological complications ranged from 24% to 50% depending on the implant system and type used in the included studies, with one study reporting more than 50% rates, which is uncommon. The differences among the included studies were attributed to the crown height and the single design of the single crown restorations, which were independent of the pre-defined protocols. Although the incidence of peri-implant mucositis could be managed surgically, it is a more serious biological complication [18].

Moreover, the rates of peri-implant mucositis could be proportional and a mediator of the implant failure in short implants across studies. In case of peri-implantitis, short implants are easily lost rather than remaining in situ, which could lead the studies to use non-splinted single crown, which facilitates mechanical oral hygiene and adherence to the care system.

The generalizability of the current meta-analysis is to some extent limited by the low number of included studies, seven RCTs, with a low number of patients, which could affect the robustness of the reported outcomes. However, we included all relevant RCTs and pooled data from all possible included patients. Second, an incomplete profile of the types of biological complications was reported. Additionally, no subgroups were performed or could be assessed. Other factors, such as the implant system, the restorations, or the variability of baseline characteristics, could hinder the robustness of the current findings.

## Conclusions

The present meta-analysis of seven RCTs and 393 patients and 474 implants found that short implants were associated with significant reductions of marginal bone loss and rates of biological complications, which might offer an alternative grafting material to standard long implants and sinus floor elevations. Further RCTs with long-term follow-up durations are needed to draw a more robust conclusion.
